# Fabrication and Evaluation of Poly(2-hydroxyethyl methacrylate)/Eudragit L-100 Hydrogels with Fusidic Acid to Promote Eczema Treatment

**DOI:** 10.1021/acsomega.3c02833

**Published:** 2023-07-22

**Authors:** Rukiye Miray Çınar Koyuncu, Nil Acaralı

**Affiliations:** Department of Chemical Engineering, Yildiz Technical University, Davutpasa Campus, Esenler, 34220 Istanbul, Turkey

## Abstract

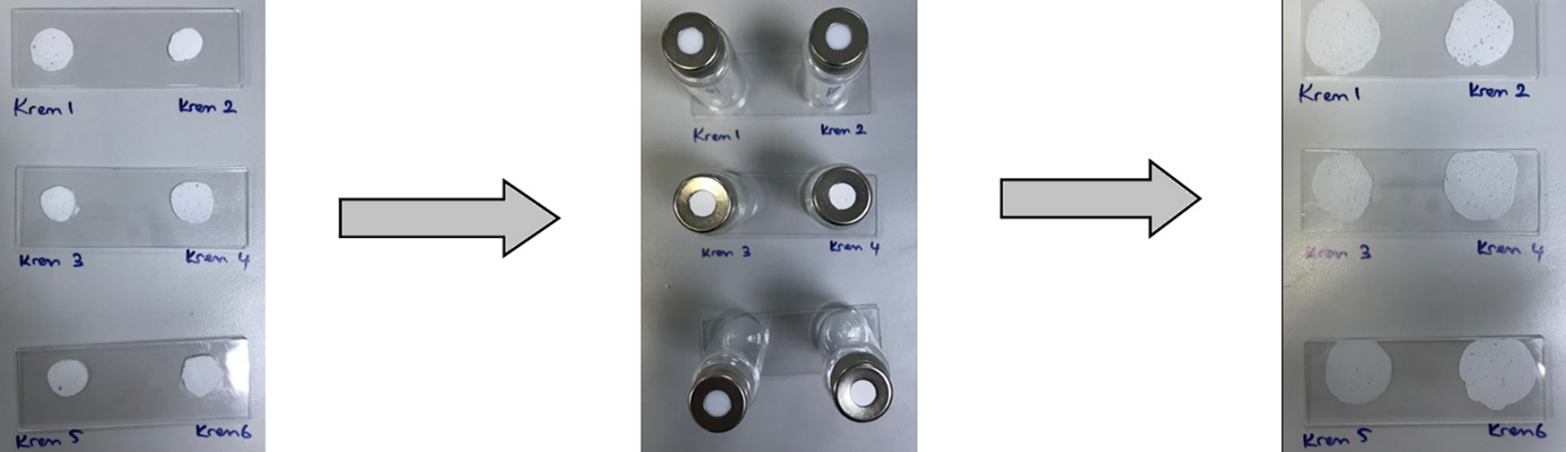

In this study, hydrogels containing 2-hydroxyethyl methacrylate (HEMA), Eudragit L-100, and fusidic acid in different compositions were prepared with the confinement method by using ammonium persulfate (APS) as a chemical initiator and ethylene glycol dimethacrylate (EGDMA) as a cross-linker to determine the most suitable formulation for use in eczema treatment. Fusidic acid (FA)-confined *p*HEMA/Eudragit L-100 in synthesis of the hydrogel was used with the Taguchi method, and the optimum synthesis conditions were determined. The swelling percentages of the hydrogels were calculated in different pH environments and distilled water. Also, the cream formulations developed and contained in chitosan and HEMA-based polymers were synthesized. Viscosity and pH values changed between 30,000 and 100,000 cP and between 5 and 6 for different cream formulations with various conditions, respectively. Also, swelling percentages of hydrogels were between 20 and 40. Fourier transform infrared spectroscopy (FTIR) and scanning electron microscopy (SEM) analyses were performed to characterize the hydrogel structure and the cream formulations. In addition, the stability of the formulations to 28 days and the changes in parameters such as appearance, centrifugation, pH, relative density, viscosity, spreading were evaluated, comparatively.

## Introduction

Hydrogels refer to a type of polymer that has a unique ability to absorb and retain significant amounts of water. Keeping the wound exudated together with foreign bodies such as bacteria, they reduce loss of fluid on the wound surface and support fibroblast proliferation.^1^ They are polymers that can absorb high rates of water or biological fluids and are cross-linked by chemical or physical bonds.^2,3^ Chitosan is a biocompatible, bioadhesive, and non-allergic biodegradable polymer.^4^ It is obtained from chitin, which is the second most abundant biopolymer in nature after cellulose.^5^ Its antimicrobial activity and ability to form composites with other materials help in cell adhesion and proliferation. It is also effective in strengthening the mechanical properties according to the place or tissue used.^6^ Another feature of chitosan is its effect on wound healing. Research has been done on the healing of wounds of chitosan for many years, and positive results have been encountered. This effect on wound healing is related to its ability to form a polyelectrolyte complex with heparin (−charged).^7^ In addition, it was reported in the literature that hydrogels made of chitosan did not have any adverse effects on people allergic to sea creatures such as crab and shrimp.^8^ HEMA is a frequently used biocompatible monomer due to its high mechanical strength and resistance to chemical and microbiological degradation.^9^ HEMA copolymers exhibit excellent biocompatibility with high water absorption. Other biomedical applications for HEMA-based materials include light microscopy. The high molecular weight HEMA homopolymer is hydrophilic and generally soluble in water.^10^ Fusidic acid is a bacteriostatic antibiotic with a steroid structure. It is one of the active ingredients used to treat bacterial infection that could be seen in skin diseases such as eczema.^11^ In literature, creams have been successfully developed for skin hydration and improved dermal drug delivery with usage of biocompatible substances.^12^ Eudragit types are non-biodegradable, non-absorbable, and non-toxic substances. The anionic Eudragit L-100 melts at pH >6 and is used for enteric coating.^13^ Creams are very broad-purpose cosmetic products that could be used externally in all parts of the body and undertake different tasks according to their function.^14^ Researchers have conducted studies to evaluate the properties of a new topical formulation consisting of chitosan gel containing 1% silver sulfadiazine as an alternative for the treatment of wounds. The new formulation demonstrated the advantageous properties and efficient release of the drug.^15^ Chitosan plays a very important role in the wound healing and various processes such as activation of fibroblasts and macrophages, giant cell migration, stimulation of polymorphonuclear cells, and collagen synthesis. In addition, it has been observed that it has a protective effect against microorganisms by showing tissue formation. In addition, it has a protective effect against microorganisms and simulates tissue formation. Due to the restorative effect of chitosan, it was stated that it plays an important role in healing of large open wounds in experiments on animals in literature.^16,17^ It was found by a different research group that combined topical corticosteroids/antibacterials were shown to be effective in eczema treatment. A fixed combination of betamethasone valerate was found to be clinically as effective as betamethasone alone and betamethasone/neomycin.^18^ A different study in the literature reported a hydrogel-based ultra-moisturizing cream for skin moisture and improved dermal drug delivery. Various active ingredient-free formulations were prepared and subjected to an in vivo skin hydration test on a balding mouse using corneal ether.^19^ Other researchers developed a microemulsion-based hydrogel formulation for penciclovir as a topical delivery system. The results showed that compared to the commercial cream, the microemulsion-based hydrogel and microemulsion could significantly increase its penetration.^20^ In another study, the effect of sodium carboxymethylcellulose and fusidic acid on gel characterization in the development of sodium fusidate-loaded wound dressing was investigated.^21^

In this study, a hydrogel form suitable for wound healing was developed while fusidic acid was used to provide an antibacterial effect on eczema by using hydrogel and fusidic acid together. In addition, to measure the stability of the creams, it was observed that the hydrogel and hydrogel-based creams exhibited stable structures when their physical and chemical properties were compared by storing them in a short-term-stability (40 °C, 75% RH) cabinet for 28 days.

## Materials and Methods

### Materials

Eudragit L-100 (Evonik), chitosan (medium molecular weight) (Sigma-Aldrich), ammonium peroxydisulfate (APS) (Merck, 98%), 2-hydroxyethyl methacrylate (HEMA) (Fluka, 97%), ethylene glycol dimethacrylate (EGDMA) (Sigma-Aldrich, 95%), fusidic acid (Ecros FS484-M), Vaseline (Sonneborn Refined Products), liquid paraffin (Eastern Petroleum Private Limited), cetyl alcohol (BASF), Polysorbate 60 (BASF), and glycerin (Vance Bioenergy) were provided from related companies with given purities.

### Methods

#### Hydrogel Preparation

**Figure 1:**
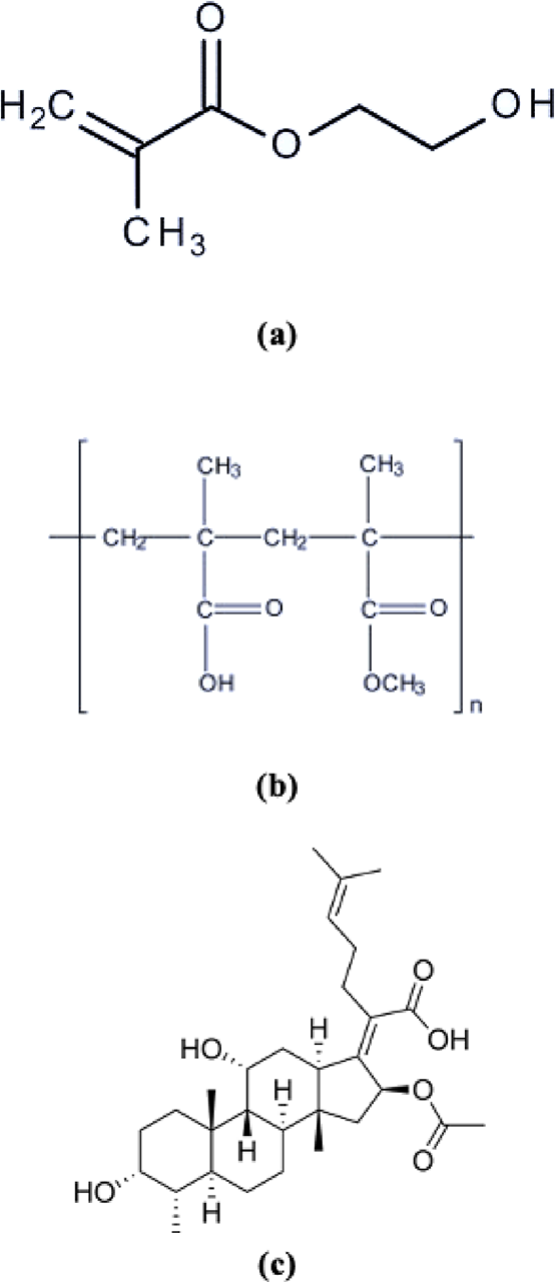
Chemical formula: (a) HEMA; (b) Eudragit L-100; (c) fusidic acid.

Eudragit L-100 (0.6–1.0 g) polymer solution and 50 mg of fusidic acid agent were added to 3 mL of ethanol and dissolved. Hydrogel-immobilized fusidic acids were synthesized homogeneously. The temperature control process was provided by mixing at 60 °C with a magnetic stirrer and a sensory control jacket at 500 rpm. To adjust the pH of the hydrogel formulation for the eczema cream, a suitable buffer system was added. The specific buffer system chosen would depend on the desired pH range and compatibility with the other ingredients in the formulation. In this way, fusidic acid was loaded into the hydrogel (Figure 1) during production by the entrapment method. The produced hydrogels were poured into glass tubes and left to dry at room temperature. Experimental design was applied for three levels and three parameters using the Taguchi method (L9 orthogonal array). The hydrogels were kept in the glass tube until gelation occurred, and then the glass tubes were broken by keeping them in an oven at 37 °C for 24 h to reach constant weight. It was put into watch glasses, and the swelling values (%) in different pH environments and water were followed.

#### Preparation of Cream Formulations

Polysorbate 60 was used as an emulsifying agent and thickener when preparing cream samples; glycerin as a moisturizer and emollient, liquid paraffin as an oil carrier; Vaseline as an emollient, thickener, and ointment base; and cetyl alcohol as a softener, viscosity agent, and emulsifier. Creams are emulsion forms that are formed by dispersion of insoluble substances such as water and oil with the help of a third substance with emulsifying properties.

#### Preparation of Chitosan-HEMA Hydrogels

Chitosan in different proportions was mixed with 2% acetic acid solution in a magnetic stirrer until dissolved. Different amounts of HEMA were added to the chitosan solution at different rates and mixed until it became homogeneous. For the preparation of the water phase, the prepared chitosan–HEMA hydrogel was added by mixing distilled water and glycerin. The pH value was adjusted between 4.5 and 6.0 with 10% (w/v) NaOH solution. A fusidic acid active substance was added on it and mixed and then homogenized. For the preparation of the oil phase, Vaseline, liquid paraffin, cetyl alcohol, and Polysorbate 60 were mixed and heated to 80 °C. When a clear mixture was obtained, the mixture was stirred at low speed and cooled to 40 °C.

In the preparation stage of the final mixture, the obtained oil phase and water phase were mixed at 40 °C and turned into a homogeneous mixture. This mixture, which became a cream, was homogenized again. Afterward, it was mixed with a mechanical mixer at low speed and cooled to room temperature. With the help of a homogenizer, the phases were homogenized within each other (water phase:oil phase = 2:1) (Table 1).

**Table 1:** Chitosan, HEMA Amounts, and Excipients in the Oil Phase in Cream Formulations

	chitosan (mg)	HEMA (mL)
cream 1	300	15
cream 2	300	
cream 3	600	15
cream 4	300	30
cream 5		15
cream 6		

oil phase	mass ratio
Vaseline	1
liquid paraffin	2
cetyl alcohol	2
Polysorbate 60	1

#### Characterization

Fourier-transform infrared spectroscopy (FTIR) (Bruker, Alpha) analysis is a widely used technique for the characterization of cream formulations. It provides information about the functional groups present in the cream, allowing for identification and qualitative analysis of various components. The measurement range in wavelength is between 500 and 4000 cm^–1^. SEM (scanning electron microscopy) (Zeiss EVO LS 10) testing was carried to examine and evaluate the samples in high resolution in detail. The samples were magnified by scanning with an electron beam. It was coated with gold/palladium (Au/Pd) to prevent glare during imaging of the sample. Images were taken from the samples in four different sizes as 500x, 1.0 Kx, 5.0 Kx, and 10.0 Kx.

While the characterization of the hydrogels was found by calculating the % swelling efficiency in different pH environments and water, FTIR spectrophotometry and SEM analyses were also performed and their morphology was examined. The characterization of the cream samples, on the other hand, was examined by performing the appearance, centrifugation, temperature, pH, viscosity, spreadability, relative density, FTIR spectrophotometry, and SEM analyses. Although many methods have been used to measure the swelling degree of the hydrogel, the most used method was calculated using eq 1. It is a measurement with weighing before and after inflation and finding the ratio by using weight. The swelling efficiency (%) of hydrogels placed in pH = 2, pH = 4, pH = 9, and pH = 11 and pure water environments was calculated according to eq 1:
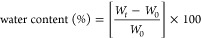
1

In eq 1, *W_t_* and *W*_0_ represent the swollen weight of the hydrogel and the dry weight of the hydrogel, respectively. The appearance control of the cream samples was done visually. With the centrifugation process, no phase separation was expected in the cream samples. To see the physical stability of the different cream samples, the samples were kept in beakers with their mouths covered with parafilm, and it was visually checked that no phase separation occurred. Then, to check their physical stability under forced conditions, they were subjected to centrifugation and their homogeneity was visually compared. All cream samples were centrifuged at 5000 rpm for 30 min.^22^ An accelerated stability study was exerted to evaluate the stability of the cream formulations. Stability of the cream was monitored by exposing it to high-temperature and humidity conditions (40 ± 2 °C/75% RH ± 5) for parameters such as appearance, pH, viscosity, and density. In addition, their physical stability was investigated during the 28-day stability period by keeping them at 25 ± 2 °C/60% RH ± 5) long-term stability conditions and comparing with the literature.^23^ Before starting to measure the samples, the pH meter was calibrated using standard buffer solutions. 15–20 g of cream sample brought to 25 °C was taken into a beaker, the electrode of the pH meter was immersed in the sample cup, and the measured value was recorded. The pH range of a healthy human skin varied between 4 and 6 in the literature. Therefore, formulations intended to be applied to the skin were produced in accordance with pH values close to this range. In addition, the pH value of the emulsion was also used to monitor its stability. It was known that the viscosity of an emulsion is critical during application for many reasons. It gives information about the flow properties of the emulsion, such as ease of application, spreadability, and the feeling it leaves on the skin. The viscosity of the continuous phase was important because of its effects on the agglomeration and/or creaming rate. Therefore, the viscosity of an emulsion is an important factor in determining its stability.^24^ The viscosity of the sample was determined with a 12–15 g sample using a Brookfield viscometer, LVDV-II+, SSA, spindle 25 at 4 rpm. The measurement was repeated three times, and the average value was calculated (cP). The spreadability of the cream placed between two slippery slides under a certain load was calculated in terms of area. Two sets of standard-sized glass slides were taken, and the cream formulations were placed on the slides in equal mass.^25^ The other glass slide was placed on top of the cream formulations so that the cream was sandwiched between the two slides, and an equal amount of approximately 17 g of GC vials by weight was placed on the top slides. After 1 min, the weight distribution areas were calculated. Distribution areas were calculated using eq 2:
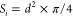
2where *S* is the spreading area due to applied mass (mm^2^) and *d* is the mean diameter (mm) obtained from the spread of the sample.^26^

For relative density measurement, a clean and dry pycnometer bottle was weighed and the empty weight of the bottle was noted (*P*_0_). The pycnometer was filled with the sample, and it was waited for the sample to reach a temperature of 25 °C. The excess sample was removed by wiping the pycnometer with a dry cloth (*P*_c_). The pycnometer was thoroughly cleaned and filled with freshly boiled and cooled water. The temperature of the pycnometer was set to 25 °C. The relative density value of the cream was calculated using eq 3. Care was taken not to leave any air bubbles while the cream was placed in the pycnometer.
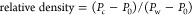
3where *P*_c_, *P*_0_, and *P*_w_ are the weight of the pycnometer when filled with cream (g), the weight of the pycnometer when unloaded (g), and the weight of the pycnometer when filled with water (g), respectively.

Fourier transform spectroscopy is an analytical method that measures the infrared intensity of light versus the wavenumber. The vibration movement of the molecule ensured the absorption of the IR rays.^27^ SEM was generally used to determine the morphology and mineralogy of natural resources and chemical composition of the materials. In this study, the morphological properties of the cream and hydrogel samples were examined by the SEM technique by first drying them in an oven at 40 °C and then without needing for coating and carbon tape coating.

## Results and Discussion

### Calculation of Swelling Efficiency (%) of Hydrogels

Swelling efficiency, also known as swelling capacity or hydration capacity, refers to the ability of a substance or material to absorb and retain moisture. In the context of eczema treatment, swelling efficiency plays a significant role. Proper moisturization is essential to alleviating the symptoms and improving the condition of the skin. Substances with a high swelling efficiency could absorb and retain moisture, providing prolonged hydration to the affected areas. Therefore, it was thought that it helps restore the skin barrier and reduces dryness. Swelling efficiency could also be beneficial when it comes to delivering medications or topical treatments for eczema. Materials with high swelling capacity could absorb and retain active ingredients, allowing for better penetration into the skin. The high swelling capacity ensures that the medication stays in contact with the affected area for a longer time, increasing its effectiveness. HEMA is a monomer that is widely utilized in the pharmaceutical industry for applications. HEMA is known for its excellent biocompatibility, meaning it is well-tolerated by living tissues and does not cause significant adverse reactions. This situation makes it suitable for use in contact with the human body, such as in drug delivery systems. Also, HEMA possesses hydrophilic properties. This property is beneficial in drug delivery systems where controlled release of the drug is desired. HEMA-based hydrogels or polymer matrices could absorb and retain water, allowing for the controlled release of drugs over time. Another reason for their preference is their ease in undergoing polymerization, forming a stable polymer network. This enables the synthesis of HEMA-based polymers with tailored properties, such as mechanical strength, flexibility, and porosity. HEMA-based polymers are typically stable and resistant to degradation under normal physiological conditions. This stability also ensures that the pharmaceutical formulations maintain their integrity and functionality over a desired period. Compatibility with other monomers is another important reason for preference. HEMA could be copolymerized with other monomers to achieve specific properties. This versatility allows for the customization of polymer formulations to suit different pharmaceutical applications.

In experimental studies, to reach the right result, it is necessary to design the appropriate experiment, to determine the parameters correctly, and to calculate what was expected from the result. This situation causes the work to take a long time, increasing the cost and effort. It is possible to examine the experimental designs made to prevent this into two groups.^28^ The Taguchi method is an optimization method used for designing of high-quality systems. This method provides a systematic approximation to optimizing performance and quality designs. Uncontrollable effects that would negatively affect the quality of the product during production are determined by this method. One of the basic concepts frequently used at this stage is signal/noise analysis (S/N: signal/noise). In this optimization study, the swelling efficiency (%) values of the hydrogels, whose swelling was examined in different pH environments, were calculated^29^ (Table 2). The method also uses orthogonal array, which is an efficient and balanced design, to explore a limited number of experiments while providing meaningful information about the factors influencing the outcome. This reduces the number of experiments required compared to traditional one-factor-at-a-time approaches. The Taguchi method also introduces the concept of the signal-to-noise ratio to evaluate the performance of a product or process. The S/N captures the variability in the output and classifies it into three categories: the smaller-the-better, the larger-the-better, and the nominal-the-best. By optimizing the S/N, the performance and robustness of the product or process are improved. The swelling behavior of hydrogels, including their swelling efficiency, could be influenced by the presence of different functional groups, such as carboxyl groups. These functional groups could influence the pH-based swelling behavior of hydrogels. The presence of carboxyl groups in a hydrogel introduces ionizable acidic functionalities. When the pH of the surrounding environment changes, the ionization state of the carboxyl groups could also change. This change in ionization affected the swelling behavior of the hydrogel. The presence of positively charged protons reduced the repulsion between polymer chains, leading to a more compact structure and reduced swelling. This was due to the increased electrostatic interactions between the protonated carboxyl groups and the surrounding water molecules. The repulsion between the negatively charged carboxylate groups caused the polymer chains to expand, leading to increased swelling of the hydrogel. By manipulating the pH of the surrounding environment, the swelling behavior of the hydrogels containing carboxyl groups was controlled. This pH responsiveness could be advantageous for applications where pH-sensitive drug release or pH-triggered swelling is desired, such as in drug delivery systems or wound dressings.

**Table 2:** Optimization Levels, Parameters (1: Minimum; 2: Medium; 3: the Highest Levels) in the Taguchi Method, and Swelling Efficiency of Hydrogels in pH = 2, pH = 4, and Distilled Water Environments (%)

no./level	Eudragit	HEMA	EGDMA	FA
1	1	1	1	1
2	1	2	2	1
3	1	3	3	1
4	2	1	2	1
5	2	2	3	1
6	2	3	1	1
7	3	1	3	1
8	3	2	1	1
9	3	3	2	1

	*t* = 2 (h)			*t* = 4 (h)		
swelling (%) of hydrogels	pH = 2	pH = 4	distilled water	pH = 2	pH = 4	distilled water
1	25.84	25.16	26.74	29.34	28.54	27.66
2	28.24	27.60	29.22	31.66	31.88	30.80
3	28.08	28.79	27.23	30.57	32.57	28.00
4	26.04	27.02	26.23	28.60	29.28	27.09
5	26.08	25.94	27.62	29.56	31.05	29.23
6	28.91	30.10	37.90	32.87	36.80	41.71
7	23.25	24.28	25.74	25.98	25.61	26.39
8	27.90	27.02	29.30	31.06	32.15	31.84
9	28.74	29.82	28.03	32.61	35.87	28.86

**Figure 2:**
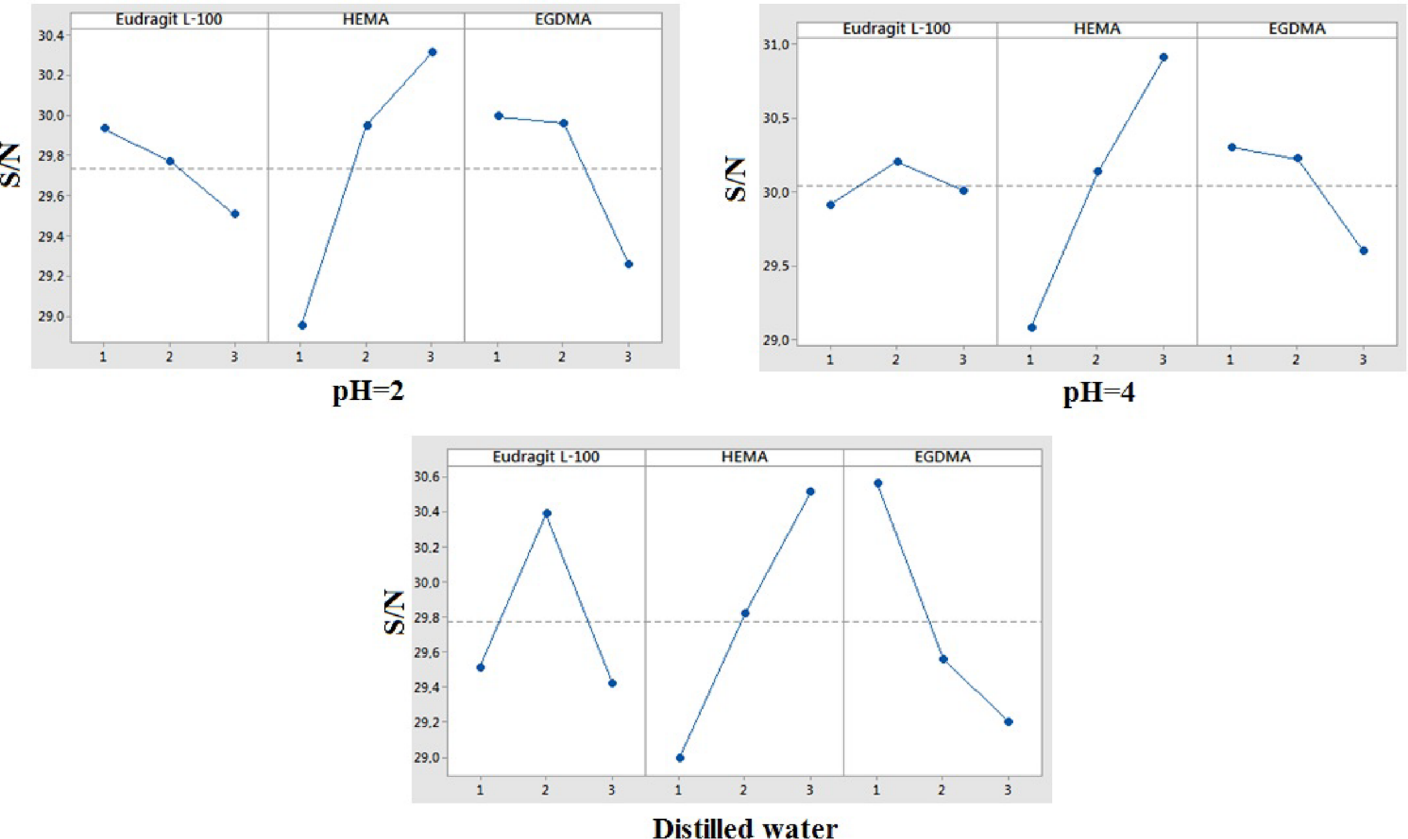
S/N ratio main effect curves for pH = 2, pH = 4, and pure water environments.

Swelling values (%) could not be calculated due to the disintegration of hydrogels in pH = 9 and pH = 11 environments. When the swelling values (%) of the hydrogels kept in a pH = 2 environment were examined, the highest swelling was seen in polymer number 6. The reason for this was the maximum amount of HEMA in its structure and the low amount of crosslinkers. Minimal swelling was seen in polymer number 7. This polymer contained minimal HEMA and maximum crosslinker and coating material Eudragit L-100. This showed that the swelling (%) of the polymer structure with increasing Eudragit L-100 and EGDMA was at a minimum level. In addition, according to the average of the S/N ratios plotted according to the % swellings of the Taguchi set, the maximum swelling was shown to be the best in the polymer where Eudragit L-100 and EGDMA were used at the minimum level and HEMA was added at the maximum level. For pH = 4, the highest swelling was observed in polymer 6. In addition, when the graph was examined, it was observed that the maximum swelling (%) increased in parallel with the increasing HEMA and decreasing EGDMA ratio in this pH environment. It was observed that the Eudragit L-100 ratio was maximum at level 2. For the swelling values (%) of hydrogels kept in a pure water environment, the highest swelling was observed in this environment and in polymer number 6, which was higher than in other media. Like in other media, polymer 7 exhibited minimal swelling. In a graph drawn from the Taguchi set, it was shown that the % swelling was parallel with the increasing HEMA and decreasing EGDMA ratio in parallel with the other graphs. In this environment, it was shown that the maximum % swelling was higher in polymers synthesized by placing the Eudragit ratio at level 2 (Figure 2). The cream had a bright-white color, soft consistency, homogeneous, and smooth structure. It also had a characteristic odor originating from HEMA in cream 1, cream 3, cream 4, and cream 5 samples. At the end of 28 days, no change was observed in its appearance and odor under high-temperature and humidity conditions of 40 ± 2 °C/75% RH ± 5 and 25 ± 2 °C/60% RH ± 5 long-term stability conditions. No phase separation was observed at the start time in the cream samples, which were centrifuged at 5000 rpm for 30 min.

After 28 days of stability, no phase separation was observed when the cream samples taken from 40 ± 2 °C/75% RH ±5 and 25 ± 2 °C/60%RH ± 5 conditions were put back into the centrifuge. The physical structures of cream samples were shown to be stable under centrifugal force. After 28 days of stability, no change was observed in the pH values of the cream samples taken from 40 ± 2 °C/75% RH ± 5 and 25 ± 2 °C/60%RH ± 5 conditions. This shows that the cream samples were stable. The viscosity of the samples, which was determined at 4 rpm using the Brookfield viscometer, LVDV-II+, SSA, spindle 25, was repeated three times, and the average results of measurements were determined. It was clearly seen that high temperature and humidity caused viscosity change in samples. Considering the results of cream 5, HEMA alone was unstable even at a low temperature. When the ratio of chitosan and HEMA increased, the viscosity of the creams increased, which might be due to better bonding of the polymers. Depending on the increasing viscosity, the relative densities of the samples also increased. The fluidity was evaluated by calculating the spreadability areas of the creams under a certain load (Table 3).

**Table 3:** pH, Viscosity, and Relative Density Values of Cream Samples

	pH			
	25 °C, 60% RH		40 °C, 75% RH	
	*t* = 0	*t* = 28 days	*t* = 0	*t* = 28 days
cream 1	5.43	5.41	5.43	5.38
cream 2	5.57	5.61	5.57	5.70
cream 3	5.35	5.33	5.35	5.30
cream 4	5.55	5.57	5.55	5.49
cream 5	5.37	5.46	5.37	5.44
cream 6	5.29	5.22	5.29	5.24

	viscosity (cP)			
	25 °C, 60% RH		40 °C, 75% RH	
	*t* = 0	*t* = 28 days	*t* = 0	*t* = 28 days
cream 1	48.722	47.191	48.722	37.653
cream 2	90.487	81.438	90.487	53.846
cream 3	80.200	77.943	80.200	119.667
cream 4	92.116	96.583	92.116	121.000
cream 5	73.528	52.790	73.528	39.365
cream 6	60.837	63.766	60.837	68.904

	relative density			
	25 °C, 60% RH		40 °C, 75% RH	
	*t* = 0	*t* = 28 days	*t* = 0	*t* = 28 days
cream 1	0.91	0.91	0.91	0.91
cream 2	0.94	0.94	0.94	0.93
cream 3	0.97	0.97	0.97	1.01
cream 4	0.91	0.92	0.91	1.06
cream 5	0.95	0.94	0.95	0.93
cream 6	0.92	0.92	0.92	0.94

### FTIR Spectroscopy of Hydrogels

**Figure 3:**
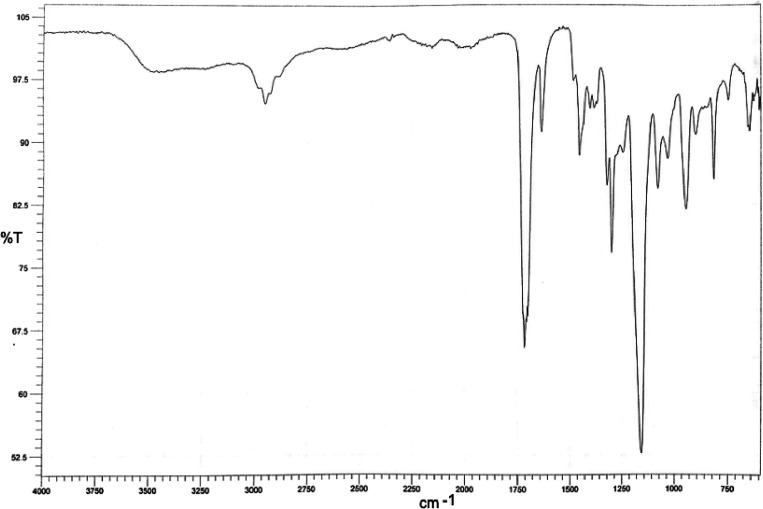
Superimposed FTIR spectrum of a produced polymer.

The peaks observed in the range of 4000–1300 cm^–1^ represented the peaks belonging to the functional groups contained in the molecules. Examination of this region of the spectrum revealed the functional groups contained in those molecules. For this reason, this range was called the distinct functional group region. The peaks seen in the range of 1300–400 cm^–1^ were very affected by the structure of the molecules. All the peaks seen in this range were like a fingerprint of the molecule, being specific to the molecule under investigation. For this reason, this region was called the fingerprint region and it was not easily understood to which vibration the peaks observed in this region belong.^30^ As clearly seen in Figure 3, all polymers were identical in structure. However, there was a difference in their peak intensities. This caused different tendencies and stresses due to the addition of the monomer, crosslinker, and initiator materials in the polymer structure at different rates. It was due to broadband O–H vibrations between 3700 and 3000 cm^–1^. The absorption peaks in the 3000 and 2850 cm^–1^ bands were caused by the vibrations of the −CH groups.

While fusidic acid had characteristic peaks in the form of two different peaks at 1686 and 1748 cm^–1^, it showed that it turned into a single sharp peak in this range with the effect of monomers in the structure and substances such as initiator and crosslinker and polymerized with the structure. In the same range, the characteristic peak from Eudragit and the characteristic peak from HEMA were added to the structure of this sharp peak. This showed that all substances in the structure were polymerized. The vibrations on this sharp peak were due to C=O ester bonds. An asymmetric methyl slope (CH_3_) was seen in the 1440–1470 cm^–1^ band, while the sharp peak at 1163 cm^–1^ belongs to the stretching of the C–O bond. The peak seen at 750 cm^–1^ was seen in all polymers and had different peak intensities due to the different amounts of HEMA added in the structure.^31^

The disappearance of the vinyl band in monomers was evaluated by using FTIR analysis. The characteristic wavenumber range was determined to identify the vinyl band or peak associated with the vinyl band in the monomers. The vinyl band typically appears in the region between 1600 and 1650 cm^–1^, corresponding to the C=C stretching vibration. If the vinyl band completely disappeared or significantly decreased in intensity in the post-reaction spectra compared to the baseline spectra, it suggested the potential disappearance of the vinyl functional group. The absence or reduction of the peak indicated that the C=C double bond had undergone a reaction or conversion. In addition to the disappearance of the vinyl band, it was important to examine the entire spectrum for any other changes. By comparing the FTIR spectra of monomers before and after a reaction or process, the disappearance or significant reduction in the intensity of the vinyl band provided evidence of chemical transformations involving the vinyl functional group.

**Figure 4:**
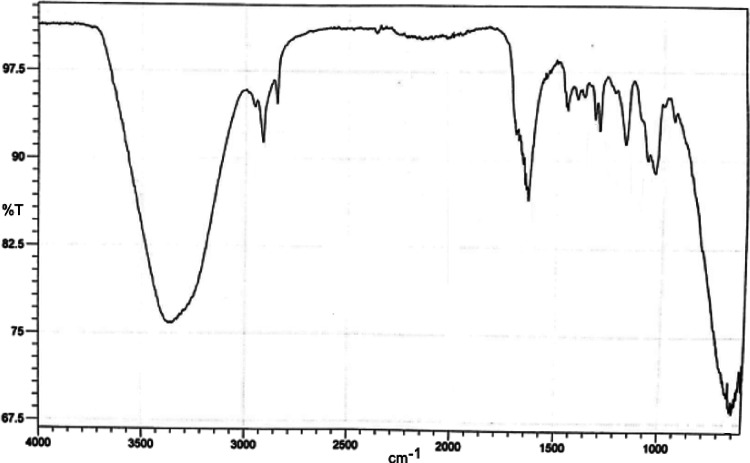
Superimposed FTIR spectrum of the cream sample.

In Figure 4, the similarity of vibrations was compared by overlapping six different cream samples. When the samples were examined, the peak intensity of the peaks was different in each spectrum, which was due to the use of different ratios of chitosan and HEMA. Cream 1, cream 3, and cream 4 sample contained different proportions of chitosan and HEMA. Cream 2 contained only chitosan, cream 5 only contained HEMA, and cream 6 did not contain chitosan and HEMA. The wide peak in the 3700–3000 cm^–1^ band was the water peak caused by O–H vibrations. Small peaks seen at 2800–3000 cm^–1^ were caused by C–H vibrations with the presence of aliphatic hydrocarbons.^31^

The peak observed at 1698 cm^–1^ was seen in all cream samples except cream 2. While it was in the form of vibration in cream 6 and cream 3, it was more prominent in cream 4 and cream 5. Also, at this point it was in the form of a sharp peak in the cream 1 sample. While this peak, which was the specific peak of HEMA, was seen at 1714 cm^–1^, this point shifted in the cream samples due to the bonds formed between them due to the characteristic peak of the fusidic acid in the structure at 1686 cm^–1^.

In addition, one of the characteristic peaks of fusidic acid seen from 1648 cm^–1^ disappeared and it was observed that it was well bonded with the structure in all cream samples. The peak at 1633 cm^–1^ was clearly seen in all cream samples. The peak seen at 750 cm^–1^ shifted in all cream samples, indicating that HEMA was well fused with the structure. Considering the peak intensity and the presence of different groups, it was observed that the structure of the best cream 1 was well fused. Also, the results were compatible with literature.^32,33^

### SEM Analysis of Hydrogels

**Figure 5:**
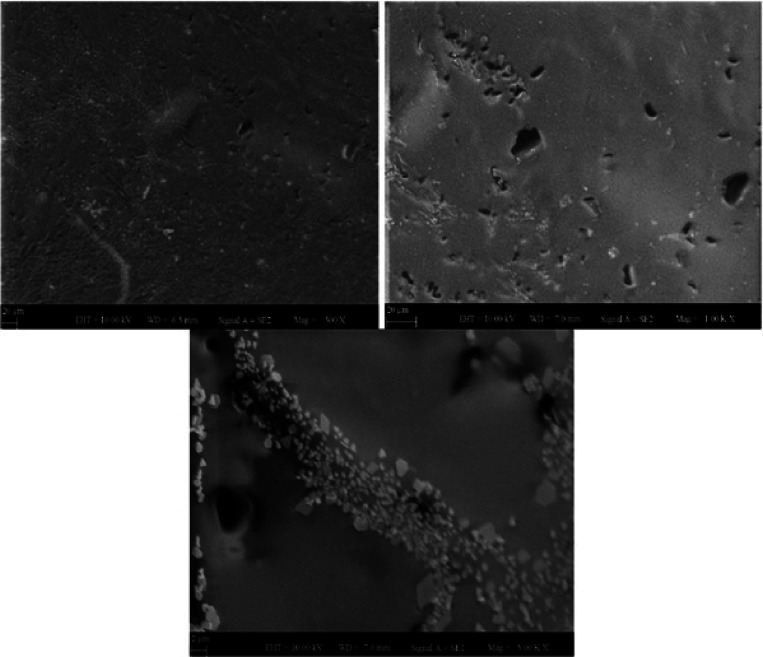
Morphological structure of polymer 6 in SEM analysis at 500, 1000, and 5000 magnifications.

SEM analysis is a technique used to create an image by scanning a sample with a focused electron beam. To ensure the conductivity of the samples for SEM analyses, morphological examination was carried out without the need for this in the hydrogel samples, while the gold plating processes were performed first. This clearly showed that the cream samples were highly conductive. In addition, imaging was performed with SEM up to 2 μm particle size. This showed that they were stable and had high strength. When the morphological structure of polymer 6, which was chosen as the optimum point, was examined, it was seen that it had a more homogeneous structure without pores. As seen in images taken from the vertical section of the hydrogel, it was seen that the hydrogels were non-porous and had a smooth structure. The roughness seen in the photo was formed during the sectioning. The reason for this was that with the increasing crosslinker ratio, the structure became more dense and tougher, forming more stable polymers (Figure 5).

**Figure 6:**
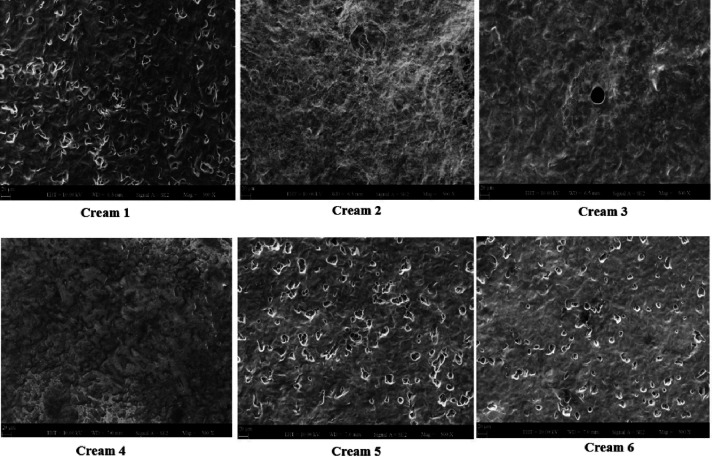
Morphological structure of SEM analysis at 500 magnifications of six different creams.

For creams, when all SEM samples were examined, it was clearly seen that the structures were homogeneously distributed, but granular structures were in some structures while in others these grains were absent. The active ingredient, fusidic acid, was added to all cream samples. In the SEM analysis for cream 1, cream 5 and cream 6, pores were smaller than 10 μm. With increasing amount of chitosan and HEMA, the three-dimensional networks formed a homogeneous structure by adhering to each other and ensuring the integration of the emulsion with each other. In addition, although pores were seen in all cream 1, cream 5, and cream 6, it was homogeneously dispersed in the emulsion. In addition, it was seen that cream 2, cream 3, and cream 4 were connected to each other with tighter bonds compared to other cream samples (Figure 6) and the results were compatible with literature.^34−40^

## Conclusions

The formulation, development, and evaluation of hydrogels to be used in the treatment of eczema were provided, and then cream formulations were developed and characterized so that they could be used in the industry. In this study, biocompatible hydrogels were successfully prepared as an alternative to drug studies in literature. A total of nine experiments were carried out by applying three levels and three parameters with the Taguchi method, and a low-cost production was achieved by applying fewer test sets in a shorter time. As the crosslink density increased, the absorption capacity of HEMA, which was a hydrophilic monomer, decreased and the % swelling efficiency remained at a lower level because a tighter structure was formed. The common feature of all sets was the maximum % swelling of polymer 6. When the characterization (FTIR, SEM) of the produced hydrogels was examined, it was seen that the successful coupling of fusidic acid, HEMA, chitosan, and Eudragit L-100 was achieved. When the SEM samples of the creams were examined, it was clearly seen that all cream samples were evenly distributed in the structure. With the increasing amount of chitosan and HEMA, the three-dimensional networks formed a homogeneous structure by adhering to each other and ensuring the integration of the emulsion with each other. It was seen that cream 2, cream 3, and cream 4 were connected to each other with tighter bonds than other cream samples. This study concluded that it was possible to develop creams containing chitosan and HEMA and could be used in the treatment of eczema. It was expected that this study would shed light on different studies to be conducted in the field of medicine and medicine in the future.

## Data Availability

The data and materials are available.

## Notes

All authors have been personally and actively involved in substantial work leading to the paper and are consent to participate.

## Notes

The paper is not currently being considered for publication elsewhere, and all authors are consent for publication.
